# Elevation of *EIF4G1* promotes non‐small cell lung cancer progression by activating mTOR signalling

**DOI:** 10.1111/jcmm.16340

**Published:** 2021-02-01

**Authors:** Ying Lu, Shanshan Yu, Guangxue Wang, Zuan Ma, Xuelian Fu, Yueyu Cao, Qinchuan Li, Zengguang Xu

**Affiliations:** ^1^ Research Center for Translational Medicine Shanghai East Hospital Tongji University School of Medicine Shanghai China; ^2^ Department of Clinical Laboratory Shanghai East Hospital Tongji University School of Medicine Shanghai China

**Keywords:** apoptosis, cell proliferation, EIF4G1, mTOR, non‐small‐cell lung cancer

## Abstract

Eukaryotic translation initiation factor 4 gamma 1 (EIF4G1), as the key component of the transcription initiation factor complex EIF4F, is significantly upregulated in multiple solid tumours, including lung cancer. However, the function and mechanism of EIF4G1 in the regulation of non‐small‐cell lung cancer (NSCLC) remain unclear. Here, using the clinical samples and the comprehensive survival analysis platforms Kaplan‐Meier plotter, we observed aberrant upregulation of EIF4G1 in NSCLC tissues; furthermore, high expression of EIF4G1 showed association with low differentiation of lung cancer cells and poor overall survival in NSCLC patients. Non‐small‐cell lung cancer cell line A549 and H1703 stably infected with EIF4G1 shRNA were used to determine the function of EIF4G1 in regulating cell proliferation and tumorigenesis in vitro and in vivo. The results demonstrated that EIF4G1 promoted the G1/S transition of the cell cycle and tumour cell proliferation in non‐small cell lung cancer. Mechanistically, EIF4G1 was found to regulate the expression and phosphorylation of mTOR (Ser2448), which mediates the tumorigenesis‐promoting function of EIF4G1. The inhibition of mTOR attenuated the EIF4G1‐induced development and progression of tumours. These findings demonstrated that EIF4G1 is a new potential molecular target for the clinical treatment of non‐small cell lung cancer.

## 1 INTRODUCTION

Lung cancer has the highest incidence of all types of cancer and is the leading cause of cancer‐related death worldwide; non‐small‐cell lung cancer (NSCLC) accounts for approximately 80%‐85% of lung cancer cases. Every year, 1.8 million people are diagnosed with lung cancer, and 1.6 million people die from this disease.^1^, ^2^ Of these patients, only 15%‐25% of NSCLC patients have the opportunity to be treated with surgery, and even with radiotherapy or chemotherapy, little effect was achieved in 50% of the patients.^3^, ^4^ Hence, it is urgent to find new diagnostic markers and therapies for NSCLC.^5^ In recent years, increasing interest has been focused on identifying effective biomarkers for early diagnosis and therapeutic targets for NSCLC treatment; such biomarkers and targets include EGFR, KRAS, PD‐1, VEGF, BRAF, and HER‐2.^6^, ^7^, ^8^, ^9^, ^10^, ^11^ Although tyrosine kinase inhibitors (gefitinib and erlotinib) can significantly prolong the survival of advanced NSCLC patients with EGFR‐sensitive mutations, acquired resistance inevitably develops because the underlying molecular mechanisms of NSCLC progression are still not fully understood.^12^, ^13^, ^14^

Eukaryotic translation initiation factors (eIFs) are a class of proteins that are necessary for protein translation in eukaryotic cells, and these proteins ensure the correct formation of mRNA‐ribosomal complexes.^15^, ^16^ Eukaryotic translation initiation factor 4 gamma 1 (EIF4G1) was first discovered as one of the constitutive proteins of the transcription initiation factor complex EIF4F.^17^ As a key scaffold protein, EIF4G1 enables other factors associated with the initiation of translation to play their respective roles.^18^ In addition to binding to the initiator, EIF4G1 can also regulate the initiation of translation by interacting with other proteins and RNA.^19^

In recent years, EIF4G1 expression has been found to be significantly increased in a variety of tumours, such as breast cancer, ovarian cancer, prostate cancer, multiple myeloma and nasopharyngeal carcinoma, and EIF4G1 plays a central role in promoting tumour angiogenesis, malignant transformation and phagocytosis during tumour progression.^20^, ^21^, ^22^, ^23^, ^24^ Our previous results showed that ubiquitin‐specific protease 10 (USP10) interacted with EIF4G1 and negatively regulated NSCLC cell survival.^25^ As a tumour suppressor gene, USP10 was found to reverse the effect of Mdm2 on the exocytosis and degradation of p53.^26^ However, the role of EIF4G1 in NSCLC, the relationship between EIF4G1 and the clinicopathological characteristics of NSCLC and the underlying mechanisms remain unclear.

In this study, we investigated the role of EIF4G1 in the pathogenesis of NSCLC and examined its correlation with the clinicopathological characteristics of NSCLC. We identified the effects of EIF4G1 on tumour cell growth and in vivo tumorigenesis, confirmed the increased expression levels of EIF4G1 in primary NSCLC tissues, and observed a positive correlation between the levels of EIF4G1 and the progression of NSCLC. Furthermore, we showed that mTOR signalling was required for EIF4G1‐mediated NSCLC progression.

## 2 MATERIALS AND METHODS

### 2.1 Patient samples and ethics approval

One hundred and twenty‐eight human NSCLC samples and adjacent normal tissues were obtained from patients undergoing surgical resection at Shanghai East Hospital of Tongji University. No patient received preoperative chemotherapy or radiotherapy. Informed consent was obtained from each patient. The whole study was approved by the Ethics Committee of Shanghai East Hospital.

### 2.2 Cell lines and reagents

Human NSCLC cell lines, A549, H460 and H1703 were provided by the Cell Bank of the Chinese Academy of Sciences; normal human pulmonary epithelial cell line BEAS‐2B was purchased from the American Type Culture Collection. All the cell lines were cultured in Dulbecco's modified Eagle's medium (DMEM) supplemented with 10% foetal calf serum (FBS) and incubated in a humidified chamber with 5% CO_2_ at 37°C. Rapamycin was purchased from Selleck Chemicals, US.

### 2.3 Immunohistochemistry

All the specimens were fixed in 10% neutral buffered formalin, embedded in paraffin, and cut into 5‐μm‐thick tissue sections. The tissue sections were deparaffinized and rehydrated for immunohistochemical staining. The sections were heated at 95°C for 20 minutes in Dako Target Retrieval Solution (Dako). After blocking with 3% H_2_O_2_, the sections were incubated overnight with primary antibodies (EIF4G1, CST, 8701S, rabbit; mTOR, CST, 5536T, rabbit) at 4°C. Following three washes with phosphate‐buffered saline (PBS) for 15 minutes each, the sections were incubated with a secondary antibody (mouse anti‐rabbit IgG‐HRP, Santa Cruz, sc‐2357) for 30 minutes at room temperature. Finally, after another three washes with PBS, the sections were visualized by using a diaminobenzidine (DAB) substrate kit according to the manufacturer's instructions. A semiquantitative analysis was applied using a well‐established scoring method. The staining results were classified according to the percentage of positive cells by two independent investigators. The percentage of positive cells was assigned a score of 0‐4, where 0 = no staining, 1 = 1%‐25% staining, 2 = 25%‐50% staining, 3 = 50%‐75% staining and 4 = 75%‐100% staining. The tissues with score of 3 or 4 were defined as high expression and those with score of 0, 1 or 2 were defined as low expression.

### 2.4 EIF4G1 knockdown or overexpression in NSCLC cell lines

To establish stable EIF4G1 knockdown NSCLC cells, we used EIF4G1‐shRNA1, EIF4G1‐shRNA2 and a negative control (NC)‐shRNA as a control (Dharmacon) in A549, H1703 or BEAS‐2B cells. The EIF4G1‐shRNA or control plasmids were co‐transfected with packaging plasmids into 293T cells using Lipofectamine 2000 Reagent (Invitrogen, 11668‐019) according to the manufacturer's instructions. Forty‐eight hours after transfection, the supernatants were collected and used to treat A549 or H1703 cells. Forty‐eight hours later, the A549 or H1703 cells were selected with puromycin (1 μg/mL) and maintained in puromycin‐containing culture medium. pcDNA3.1 vector overexpressing EIF4G1 was a gift from Prof. Jian Yuan from Tongji University, which was used to transfect A549 cells for EIF4G1 overexpression.

### 2.5 Tumorigenicity in vivo

A549 cells (1 × 10^6^) in 100 µl physiological saline (0.9% NaCl) were injected subcutaneously into the flanks of BALB/c nude mice. The appearance of tumours was monitored after 2 weeks. The largest perpendicular diameters of the resulting tumours were periodically measured, and tumour volumes were calculated according to the following formula: larger diameter × (smaller diameter)^2^/2. The animals were killed when the tumours reached the bioethically permitted limit of 2500 mm^3^. Animal survival was defined as the period after the injection of the tumour cells until the animals were killed.

The China Animal Welfare Guidelines were followed during all the experimental animal procedures. The Institutional Animal Care and Use Committee in Shanghai East Hospital, Tongji University, approved this study protocol.

### 2.6 Western blot analysis

The cells were lysed in RIPA buffer, and the protein concentration was determined by BCA assay (Beyotime Inc). The total protein (30 μg) was separated by using a 10% SDS‐PAGE gel and transferred onto a polyvinylidene fluoride (PVDF) membrane. After blocking in 5% milk (w/v) at room temperature for 1 h, the membranes were incubated with the primary antibodies at 4°C overnight with gentle agitation. β‐actin (1:1000, Santa Cruz, sc‐8432, mouse) was used as the loading control. Following 3 × 15‐min washes in PBST buffer, the membranes were incubated with the secondary antibodies (1:2000, mouse anti‐rabbit IgG, Santa Cruz, sc‐516253; anti‐mouse IgG, Santa Cruz, sc‐516181) for another 1 h at room temperature. Detection of the proteins was achieved by using the Odyssey Infrared Imaging System (Li‐COR) according to the manufacturer's instructions. All the densitometric analyses of the bands were performed using ImageJ software (National Institutes of Health). The antibodies used in this study included: EIF4G1 (1:500, CST, 8701S, rabbit); mTOR (1:1000, CST, 2983S, rabbit), Phospho‐mTOR (Ser2448, 1:1000, CST, 5536T, rabbit), GβL (1:1000, CST, 3274T, rabbit), Rictor (1:1000, CST, 2114T, rabbit); P53 (1:1000, CST, 2524S, mouse), P21 (1:1000, CST, 2947T, rabbit); caspase 3 (1:1000, CST, 9662S, rabbit), cleaved caspase 3 (1:1000, CST, 9661T, rabbit), bcl2 (1:500, Santa Cruz, sc‐7382, mouse), bax (1:500, Santa Cruz, sc‐7382, mouse); cyclin D1 (1:1000, CST, 2978S, rabbit); and fas (1:1000, CST, 4233T, rabbit).

### 2.7 RT‐PCR

The total RNA was extracted using TRIzol™ (Invitrogen), and reverse transcription was performed using a first‐strand cDNA Synthesis Kit (Takara) according to the manufacturer's instructions. Real‐time polymerase chain reaction (RT‐PCR) was performed using Premix Ex Taq SYBR Green PCR (Takara) on an ABI PRISM 7500 (Applied Biosystems) according to the manufacturer's instructions. The sequences of the primers were as follows: EIF4G1 forward, 5′‐TTGTGGATGATGGTGGCT‐3′, and reverse, 5′‐TTATCTGTGCTTTCTGTGGGT‐3′; GAPDH forward, 5′‐TGACTTCAACAG

CGACACCCA‐3′, and reverse, 5′‐CACCCTGTTGCTGTAGCCAAA‐3′. GAPDH served as the internal control. The 2^−ΔΔCt^ method was used for quantification and GAPDH was used as an endogenous control. The tissues with the value of 2^−ΔΔCt^ ≥ 3/2 were defined as up‐regulation, those with the value of 2^−ΔΔCt^ ≤ 2/3 were defined as downregulation, and those with the value between 3/2 and 2/3 were defined as stable.

### 2.8 Cell proliferation assays

Cell proliferation was determined by MTT assays. Briefly, the A549, H1703 or BEAS‐2B cells transfected with shNC and shEIF4G1, or pVEC and pEIF4G1 were plated at 1 × 10^3^ cells/well in 96‐well plates. The CellTiter 96 Aqueous One Solution Cell Proliferation Assay Kit (Promega) was used according to the manufacturer's instructions. At the end of each period, MTT reagent was added to each well and incubated for 4 hours. Then, the formation crystals were solubilized in DMSO, and the optical density (OD) value was read at 570 nm on a spectrophotometric plate reader.

### 2.9 Flow cytometry analysis of the cell cycle and apoptosis

The cell cycle and apoptosis were analysed by flow cytometry. After collecting the cells and washing them with cold PBS, a total of 1 × 10^6^ cells were fixed with 70% ice‐cold ethanol for 24 h at 4°C. The cells were resuspended in cold PBS and incubated with 50 μg/ml propidium iodide and 0.1 mg/ml RNase A at 37°C for 15 minutes. The DNA content of the A549 or H1703 cells was determined by BD FACS Calibur cytometry and analysed by ModFit LT software (Verity Software House). A549 or H1703 cells were incubated in FBS‐free medium for 24h to induce apoptosis. For the flow cytometry analysis, the A549 or H1703 cells were trypsinized and collected for the detection of apoptosis by using an Annexin V‐FITC and PI Apoptosis Detection Kit (BD Bioscience).

### 2.10 Statistical analysis

The data are presented as the mean ± SD of 3 independent experiments. The quantitative data of two groups were analysed by two‐tailed Student's *t*‐tests, and comparisons among three or more groups were conducted using one‐way ANOVA. A survival analysis was performed using the Kaplan‐Meier method. A correlation analysis was performed between EIF4G1 and mTOR using Pearson's correlation method. All the statistical analyses were performed with the statistical software SPSS 17.0 (IBM Corporation, Armonk). A *P* value less than 0.05 was considered statistically significant.

## 3 RESULTS

### 3.1 EIF4G1 is frequently upregulated in NSCLC tissues and predicts poor prognosis

**FIGURE 1:**
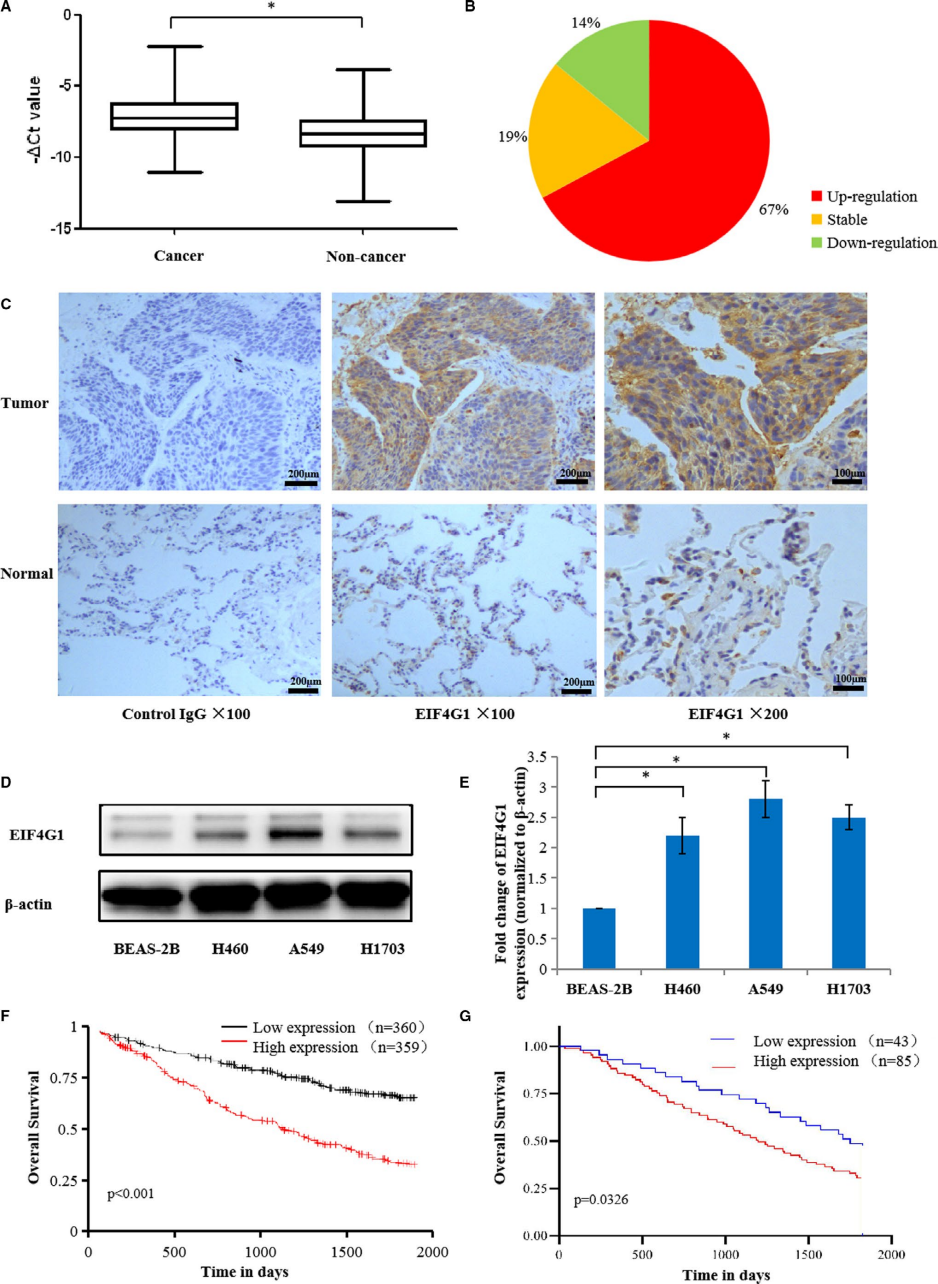
Expression pattern of EIF4G1 in NSCLC tissues and cell lines. A, EIF4G1 expression in 128 matched NSCLC cancer tissues and paired adjacent normal lung tissues was detected by RT‐PCR, and GAPDH was used as the internal control. The *P* value was calculated by Student's test. B, The pie chart shows the proportion of matched NSCLC cancer tissues with upregulated or downregulated EIF4G1 mRNA. C, The protein expression of EIF4G1 was determined by immunohistochemical staining of the NSCLC tissues with an anti‐EIF4G1 antibody, 1 bar = 200 μm or 1 bar = 100 μm. D, The expression of EIF4G1 was evaluated by Western blot in three NSCLC cell lines (H460, A549 and H1703) and the normal human pulmonary epithelial cell line BEAS‐2B. E, The relative protein levels of EIF4G1 in the NSCLC cell lines were analysed, and β‐actin was used as the internal control. F, G, Kaplan‐Meier overall survival plot comparing patients with high EIF4G1 expression (red line) and low EIF4G1 expression (black or blue line) in their tumours from Kaplan‐Meier plotter online platform (F) or 128 NSCLC patients (G). The *P* value was calculated by the log‐rank test. The data are expressed as the mean ± SD of three independent experiments. *P < .05

We first examined EIF4G1 expression in 128 paired NSCLC patient tissues by RT‐PCR. The results showed that EIF4G1 expression was significantly higher in cancer tissues than in paired adjacent normal lung tissues (Figure 1A). Among the cancer tissues, 86/128 (67%) of the samples exhibited at least 1.5‐fold increases in EIF4G1 expression, as shown in Figure 1B. In addition, immunohistochemical analysis was performed on 128 NSCLC cancer tissues and paired adjacent tissues using a specific anti‐EIF4G1 antibody. We confirmed high levels of EIF4G1 expression in the cytoplasm of NSCLC tumour cells and much lower EIF4G1 expression in the adjacent normal lung tissues from most patients (Figure 1C). Furthermore, we found that EIF4G1 expression was greatly increased in NSCLC cell lines (H460, A549 and H1703) compared to the normal human pulmonary epithelial cell line BEAS‐2B (Figure 1D,E).

To investigate the prognostic value of EIF4G1 for NSCLC, we used Kaplan‐Meier plotter, which is a web tool of comprehensive survival analysis platforms to validate survival‐associated gene expression data from NSCLC patients. (http://ualcan.path.uab.edu/index.html). ^27^ As shown in Figure 1F, EIF4G1 with higher expression correlated to poorer overall survival of NSCLC. Similar correlation was obtained in the 128 NSCLC patients tested in this research (Figure 1G).

### 3.2 Relationship between the expression of EIF4G1 and the clinicopathological characteristics of NSCLC patients

To further investigate the possible correlations between the expression levels of EIF4G1 and the clinicopathological characteristics of NSCLC patients, we analysed the clinical data of these 128 patients. The clinicopathological characteristics of the patients are summarized in **Table** **1**. We found that EIF4G1 expression was closely correlated with tumour differentiation; EIF4G1 expression in poorly differentiated tumour tissues was significantly higher than that in moderately and well‐differentiated cancer tissues, *P* < .001. We also observed that the EIF4G1 expression levels were closely correlated with lymph node involvement (N classification, N0 vs N1 + N2 + N3) and metastasis (M classification, M0 vs M1), *P* < .001. Moreover, EIF4G1 expression and clinical staging were positively correlated, and the expression of EIF4G1 in stages III and IV was significantly higher than that in stages I and II, *P* < .001. However, there was no significant correlation between EIF4G1 expression and age, gender or T classification in our analysis. The expression of EIF4G1 was significantly higher in sarcomatoid carcinoma, but there was no significant difference in other NSCLC (adenocarcinoma, squamous carcinoma, large cell carcinoma and adenosquamous carcinoma).

**Table 1:** Relationship between EIF4G1 mRNA and clinicopathologic characteristics of NSCLC (**P < .001)

Group	Number of cases	2^−ΔΔCt^	*t* or *F* value	*P* value
Age (y)				
≥65	65	4.91 ± 0.88	0.749	.455
<65	63	4.09 ± 0.65		
Gender				
Male	70	3.90 ± 0.53	‐1.208	.229
Female	58	5.23 ± 1.02		
Pathological type				
Adenocarcinoma	75	4.24 ± 0.57	24.131	<.001^a^
Squamous carcinoma	42	2.83 ± 0.46		
Large cell carcinoma	4	4.54 ± 1.75		
Adenosquamous carcinoma	3	4.55 ± 3.20		
Sarcomatoid carcinoma	4	27.04 ± 6.38		
Differentiation				
Well‐differentiated	17	0.87 ± 0.14	25.181	<.001^b^
Moderately differentiated	57	1.94 ± 0.19		
Poorly differentiated	54	8.37 ± 1.08		
T classification				
T1	12	3.20 ± 1.95	2.588	.056
T2	35	3.68 ± 0.89		
T3	60	4.09 ± 0.60		
T4	21	7.84 ± 2.18		
N classification				
N0	72	1.84 ± 0.20	‐6.288	<.001**
N1‐3	56	7.94 ± 1.07		
M classification				
M0	102	3.28 ± 0.44	‐4.794	<.001**
M1	26	9.32 ± 1.84		
Clinical stage				
I	26	1.43 ± 0.21	20.142	<.001^c^
II	47	2.08 ± 0.27		
III	36	6.07 ± 0.93		
IV	19	11.75 ± 2.43		

^a^ Compared with adenocarcinoma, squamous carcinoma, large cell carcinoma, adenosquamous carcinoma, the mRNA expression of EIF4G1 in sarcomatoid carcinoma was upregulated significantly.

^b^ Compared with well‐differentiated and moderately‐differentiated, the mRNA expression of EIF4G1 in poorly differentiated was upregulated significantly.

^c^ Compared with clinical stage I and II, the mRNA expression of EIF4G1 in clinical stage III and IV was upregulated significantly.

### 3.3 EIF4G1 promotes NSCLC cell growth and inhibits apoptosis

**FIGURE 2:**
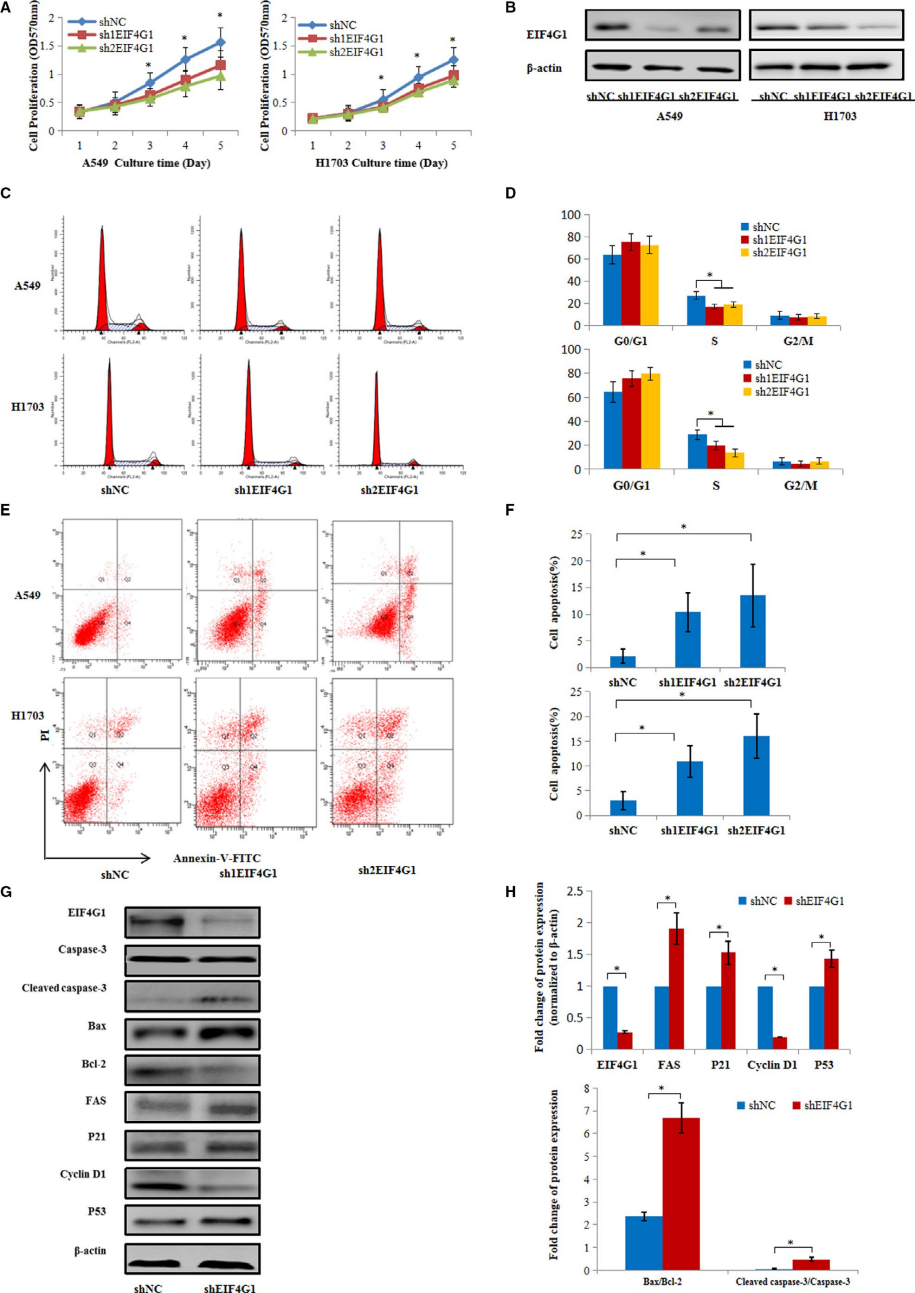
EIF4G1 promotes NSCLC cell growth. A, The growth curve of A549 and H1703 cells that were transfected with shNC or shEIF4G1, as determined by MTT assays. B, Decreased expression of EIF4G1 at the protein level in the A549 and H1703 cells treated with shEIF4G1. C, Cell cycle analysis by flow cytometry indicating the decreased G_1_/S transition of shEIF4G1‐A549 or H1703 cells treated with complete medium for 24 h after incubation in FBS‐free medium for 12 h. D, Quantification of cell cycle analysis. E, The cell apoptosis of shNC‐treated and shEIF4G1‐treated cells cultured in FBS‐free medium was examined by Annexin V/PI staining and flow cytometry analysis. F, The proportion of apoptotic cells was determined by PI‐negative and Annexin V‐positive staining. G, Western blot analysis was performed in A549 cells treated with shNC or shEIF4G1 and showed changes in the protein expression of caspase‐3, bax, bcl‐2, fas, p21, cyclin D1 and p53. H, Relative quantifications of EIF4G1, fas, p21, cyclin D1 and p53, and β‐actin were used as the internal control. The protein expression ratios of bax/bcl‐2 and cleaved caspase‐3/total caspase‐3. The data are expressed as the mean ± SD of three independent experiments. *P < .05

To determine the biological function of EIF4G1 in NSCLC, a cell proliferation assay, cell apoptosis assay and cell cycle analysis were performed on A549 and H1703 cells, which respectively represent the main pathological type of NSCLC: adenocarcinoma and squamous cell carcinoma. The MTT method was used to assess cell growth ability after silencing EIF4G1. As shown in Figure 2A,B, A549 or H1703 cells transfected with shEIF4G1 displayed significant growth inhibition compared with cells transfected with shNC, accompanied with the downregulation of EIF4G1. However, knockdown of EIF4G1 in the normal human pulmonary epithelial cell line BEAS‐2B did not show significant effect on cell proliferation (Figure S1). In addition, we measured the changes in cell cycle progression by flow cytometry analysis, and found that NSCLC cells transfected with shEIF4G1 showed a significant decrease in the S phase than those transfected with shNC (Figure 2C,D). Flow cytometry analysis was performed to determine whether EIF4G1 knockdown affects cell apoptosis induced by FBS‐free medium. Interestingly, the number of early apoptotic cells significantly increased in the shEIF4G1‐treated cells compared with the shNC‐treated cells (Figure 2E,F).

In order to further investigate the molecular mechanism underlying EIF4G1‐regulated cell growth and apoptosis in NSCLC, we detected the protein levels of growth and/or apoptosis‐related genes in A549 cells. Western blot analysis showed that EIF4G1 knockdown led to increased expression of cleaved caspase‐3, bax, fas, p21 and p53, and decreased protein levels of bcl‐2 and cyclin D1 (Figure 2G,H). These findings further suggested that EIF4G1 is required for cell viability and proliferation in NSCLC.

### 3.4 Silencing of EIF4G1 inhibits xenograft tumour growth in vivo

**FIGURE 3:**
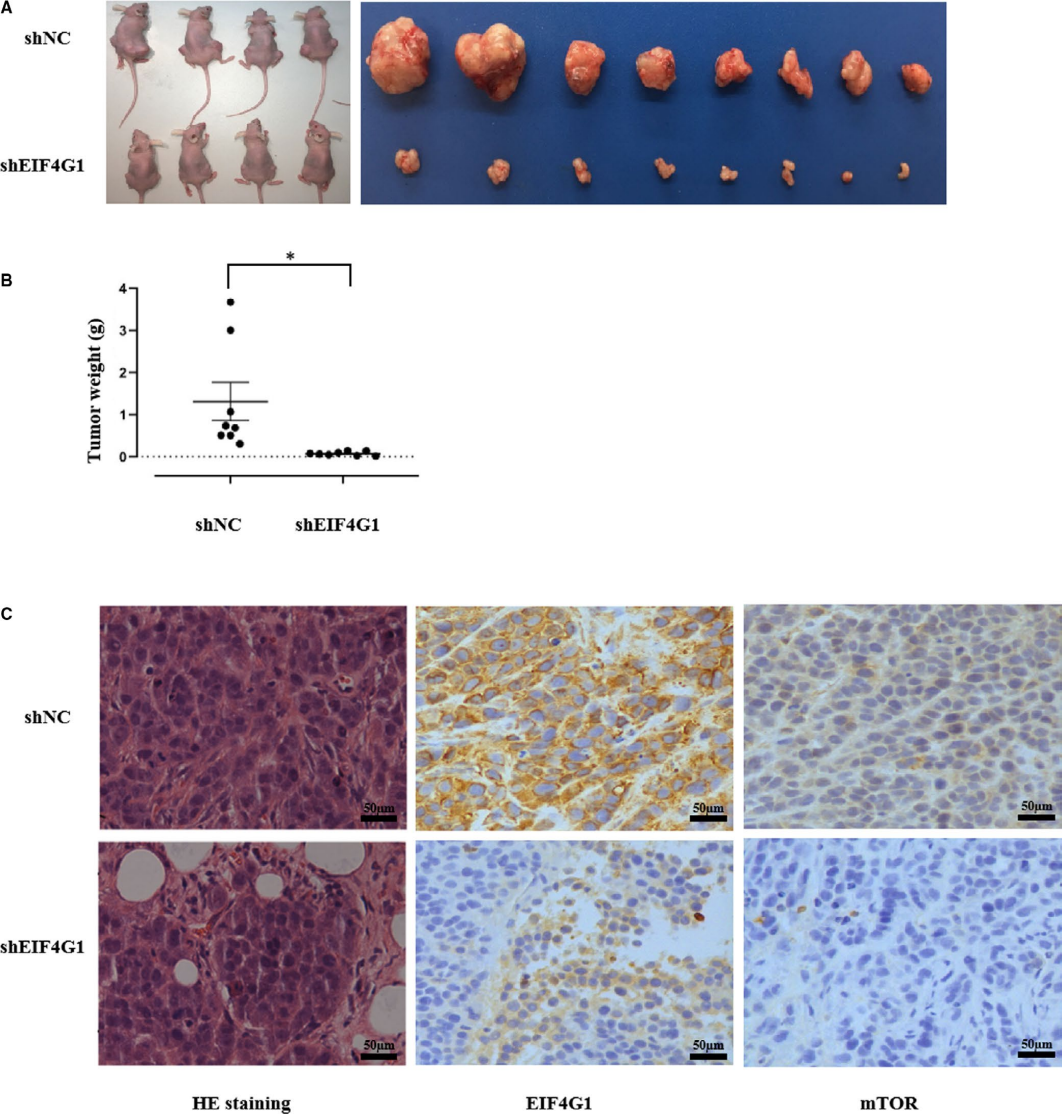
EIF4G1 knockdown suppressed the tumorigenicity of A549 cells in a xenograft nude mouse model. A, The nude mice were killed, and the tumour tissues were collected and photographed. B, The tumour weights were measured. C, Images of haematoxylin and eosin (H&E) staining and immunohistochemical staining analysis of EIF4G1 and mTOR protein expression in the tumour tissues from each group, 1 bar = 50 μm. The data are expressed as the mean ± SD of three independent experiments. *P < .05

We next assessed the function of EIF4G1 in an NSCLC xenograft mouse model. Stable EIF4G1 knockdown and control A549 cells were subcutaneously injected into nude mice (n = 8). As shown in Figure 3A,B, we found that EIF4G1 knockdown A549 cells dramatically inhibited NSCLC tumour growth in mice compared to control cells. Immunohistochemical staining confirmed that the tumours from the EIF4G1 knockdown cell‐injected mice had much lower EIF4G1 protein expression (Figure 3C). Taken together, these data suggest that EIF4G1 is also required for NSCLC tumorigenicity in vivo.

### 3.5 EIF4G1 regulates the activities of the mTOR signalling pathway

**FIGURE 4:**
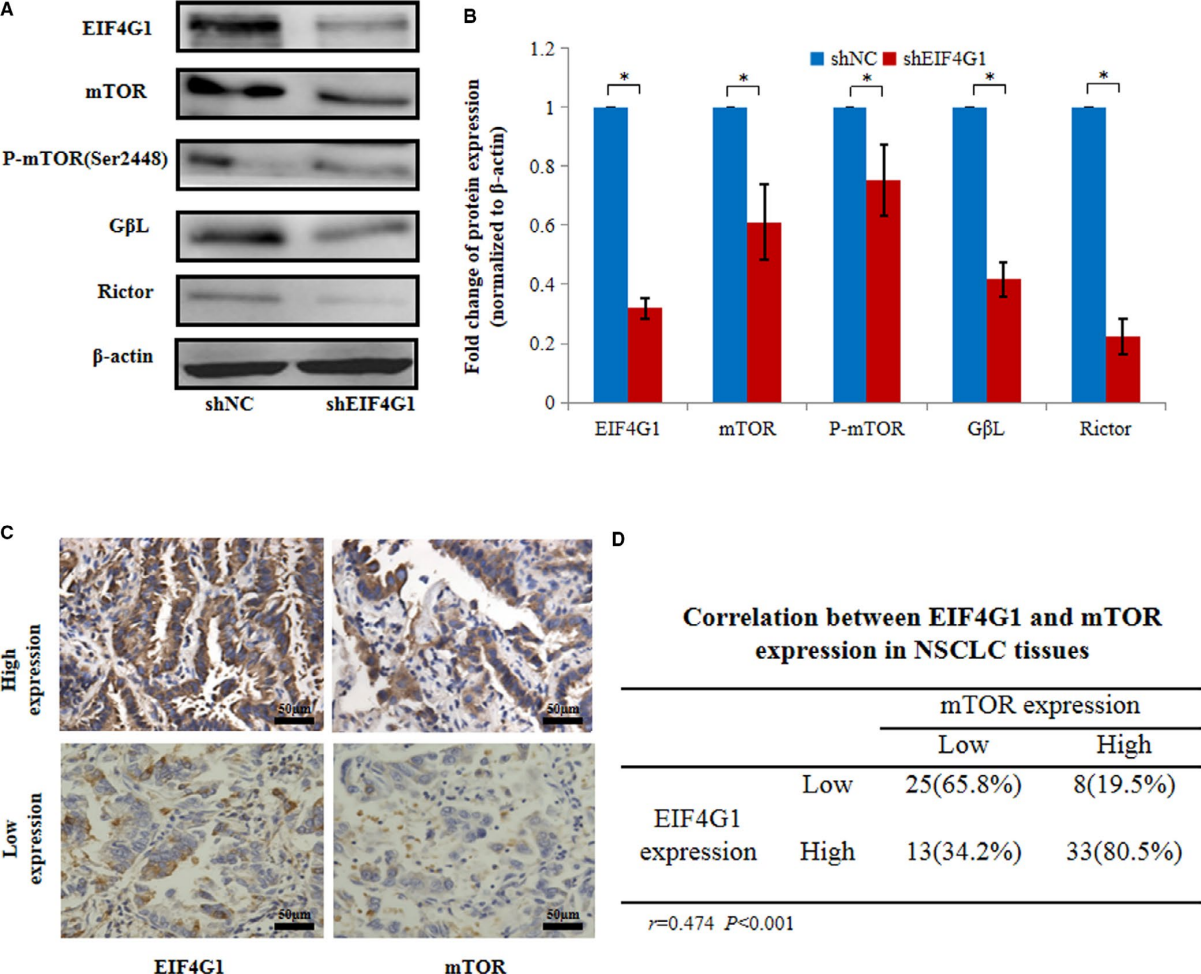
Effects of EIF4G1 on NSCLC via the activation of mTOR signalling. A, Western blot analysis was used to confirm the change in mTOR signalling in A549 cells treated with shNC or shEIF4G1. B, The relative protein levels of EIF4G1 and mTOR signalling molecules. C, Immunohistochemical staining of EIF4G1 and mTOR protein expression in the tumour tissues from NSCLC patients, 1 bar = 50 μm. D, Correlation between EIF4G1 and mTOR expression in NSCLC tissues. The data are expressed as the mean ± SD of three independent experiments. *P < .05

To determine the signalling pathway related to the EIF4G1‐mediated regulation of NSCLC cell growth, we used several Cell Signaling Technology (CST) Pathway Antibody Sampler Kits to compare the activities of common cancer‐related cell signalling pathways between the EIF4G1 knockdown and control A549 cells. We found that mTOR signalling activities were significantly affected. As shown in Figure 4A,B, the expression of total mTOR, phosphorylated mTOR (Ser2448), GβL and Rictor was downregulated in the EIF4G1 knockdown cells compared to the control A549 cells. mTOR, GβL and Rictor constitute an important signalling pathway in the regulation of tumour growth.^28^, ^29^ Next, we analysed the relationship between EIF4G1 and mTOR protein expression in NSCLC tissues through immunohistochemical analysis and showed that there was a positive correlation (*r* = .474, *P* < .001; Figure 4C,D). The same results were obtained in the xenograft tumour in vivo (Figure 3C).

**FIGURE 5:**
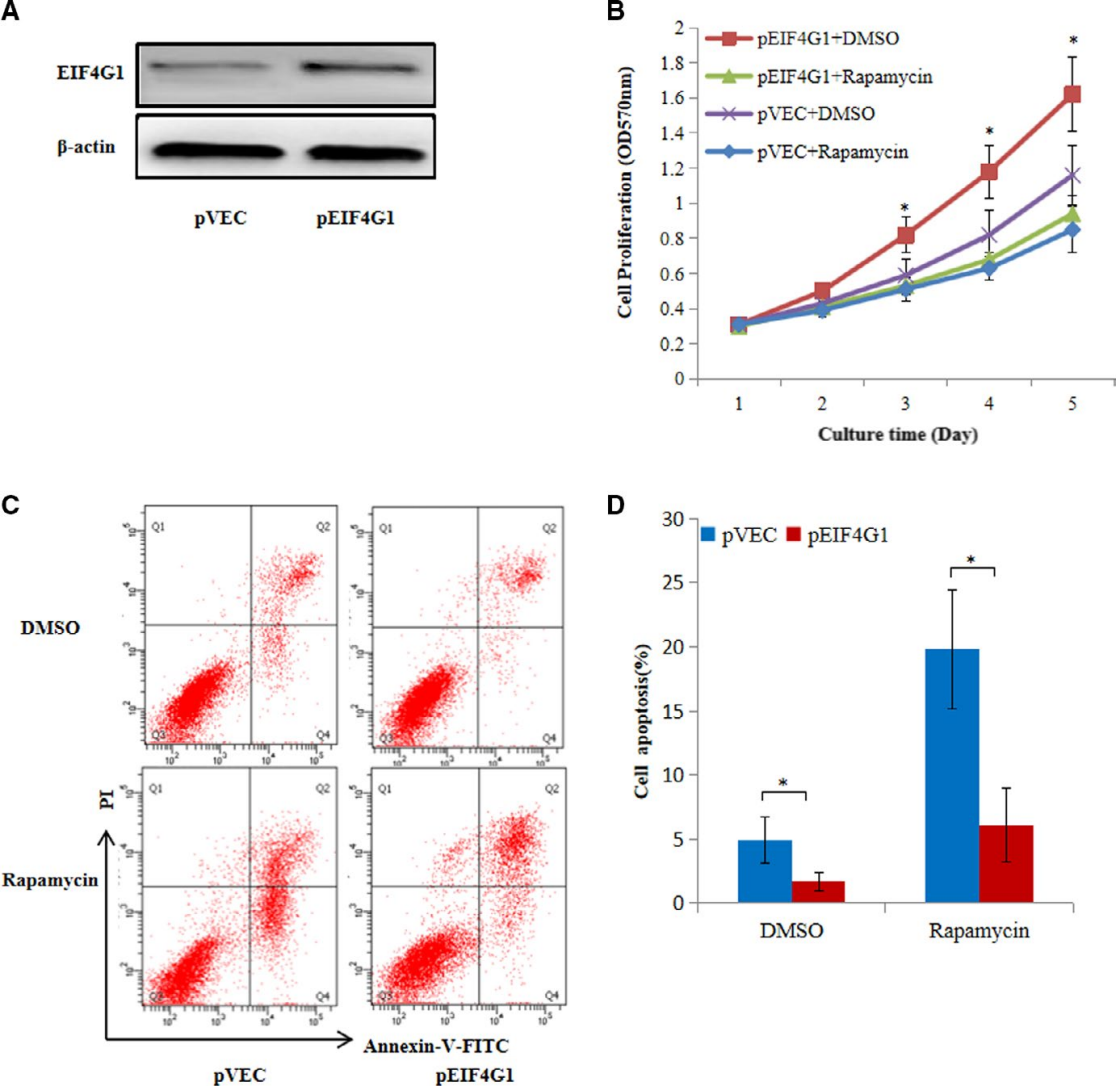
Rapamycin partially blocked the effects of EIF4G1 on A549 cell growth and apoptosis. A, Western blot analysis was performed in A549 cells treated with pVEC or pEIF4G1. B, The effects of EIF4G1 on the proliferation of A549 cells treated with DMSO or rapamycin by MTT assays. C, Cell apoptosis in pVEC‐A549 and pEIF4G1‐A549 cells treated with DMSO or rapamycin was examined by Annexin V/PI staining and flow cytometry analysis. D, The proportion of apoptotic cells was determined by PI‐negative and Annexin V‐positive staining. The data are expressed as the mean ± SD of three independent experiments. *P < .05

To address the functional relevance of EIF4G1 and mTOR signalling, rapamycin (an mTOR kinase inhibitor) was used to treat A549 cells, and its effects on cell proliferation and apoptosis were observed. As shown in Figure 5, EIF4G1 overexpression in A549 cells led to significantly enhanced growth and decreased apoptosis, which were attenuated by rapamycin treatment, demonstrating that mTOR signalling mediated oncogenic function of EIF4G1 in A549 cells. In conclusion, our results suggest that activation of mTOR signalling is required for EIF4G1‐induced tumour growth in NSCLC.

## 4 DISCUSSION

EIF4G1, as a critical component of the EIF4F complex, is required for cap‐dependent mRNA translation.^30^ The formation and development of tumours are complex processes involving multiple factors, such as some key oncogenes and tumour suppressor factors.^31^ In addition to promoting protein translation, EIF4G1 plays an important role in the initial stage of eukaryotic cell translation and is closely related to the occurrence and development of tumours.^32^, ^33^ Some studies have shown that the eukaryotic translation initiation factor EIF4G1 plays a positive role in the processes of tumour formation, proliferation and metastasis.^19^, ^20^, ^21^, ^22^, ^23^, ^24^ In the present study, we found that the mRNA and protein expression levels of EIF4G1 were upregulated in NSCLC tissues, as determined by RT‐PCR and immunohistochemistry analysis, and the expression levels of EIF4G1 were closely related to the differentiation of tumour cells; the higher the expression of EIF4G1 was, the poorer differentiated the tumour was. Further analysis of the relationship between EIF4G1 and clinicopathological variables found that the higher the clinical stage was, the higher the expression level of EIF4G1 in NSCLC was. However, there was no statistically significant correlation between the expression level of EIF4G1 and T classification, age and gender of the patient. The relationship between differentiation and high expression of EIF4G1 is our novel finding. Of the 128 cases of NSCLC, the mRNA expression of EIF4G1 in adenocarcinoma (75/128), squamous carcinoma (42/128), large cell carcinoma (4/128) and adenosquamous carcinoma (3/128) had no statistical difference, but its expression was higher in sarcomatoid carcinoma (4/128). Subsequently, targeted knockdown of EIF4G1 inhibited xenograft tumour growth in vivo. Taken together, our results suggest that EIF4G1 is closely related to the occurrence and development of NSCLC tumours.

Pulmonary sarcomatoid carcinoma is a rare, poorly differentiated subtype of NSCLC, which occupies approximately 0.1%‐0.5% of lung malignancies. Sarcomatoid carcinoma is characterized with resistance to radiotherapy and chemotherapy, resulting in high rate of local and metastatic recurrence and poor prognosis.^34^ In consistence, we herein found that high expression level of EIF4G1 is closely related with the poor differentiation in sarcomatoid carcinoma, suggesting EIF4G1 has potential to serve as a therapeutic target of pulmonary sarcomatoid carcinoma. In view of the limited number of clinical samples used in the current study, the results need to be further confirmed in the future with larger number of samples by multi‐centre prospective cohort studies, associated with long‐term follow‐up.

It was reported that EIF4G1 interacted with the ubiquitin USP10, as shown by tandem affinity purification and mass spectrometry; USP10 is located in the cytoplasm, and USP10 had been shown to be able to specifically ubiquitin and stabilize p53, thereby regulating p53‐dependent downstream function.^25^, ^26^, ^35^ To date, p53 is one of the most important tumour suppressor factors.^36^ As a transcription factor, p53 regulates a variety of cytological responses by regulating downstream target genes.^37^, ^38^ For example, p53 regulates cell cycle G1 arrest by acting on the CDK inhibitory factor p21, cell apoptosis by acting on bax, energy metabolism by acting on TIGAR of glycolysis, and autophagy by acting on AMPK. In addition to its transcription factor‐dependent function, p53 can also regulate apoptosis through protein‐to‐protein interactions. Our previous study showed that EIF4G1 combined with USP10 in the lung cancer cell line A549, as determined by co‐immunoprecipitation analysis, so we assume that EIF4G1 can inhibit the USP10‐mediated stabilization of p53 by binding to USP10, leading to the inhibition of p53 functions in cancer cells. In this study, we confirmed that EIF4G1 regulated the protein expression of p53 and other apoptosis‐related proteins, such as caspase‐3, bax, bcl‐2, fas and p21.

Up to date, the expression of EIF4G1 was up‐regulated in nasopharyngeal carcinoma, breast cancer, ovarian cancer and squamous cell lung carcinoma, but there is no report about EIF4G1 being involved in signalling pathways in NSCLC. In our study, we not only found the overexpression of EIF4G1 in NSCLC besides squamous cell lung carcinoma, but also found that EIF4G1 affects the mTOR signalling pathway in A549 cells. Knockdown of EIF4G1 with shRNA decreased the protein expression of mTOR, phosphorylated mTOR (Ser2448), GβL and Rictor. Overexpression of EIF4G1, A549 cell proliferation was inhibited by mTOR kinase inhibitor. Taken together, these data suggested that EIF4G1 regulated the protein translation of mTOR signalling molecules, which is a key pathway in control of protein translation, cell proliferation and mobility in tumorigenesis. This observation is in accordance with the role of EIF4G1 in eukaryotic cells, which was first recognized to participate in protein translation by serving as a scaffold and interacting with several other initiation factors. In our study, mTOR signalling may be downstream effectors for EIF4G1 in promoting NSCLC progression. Thus, our data implicated a new mechanism for EIF4G1 in regulating tumorigenesis and progression in NSCLC.

Up to date, several EIF4G1‐targeted molecular inhibitors have been developed and tested in treatment of cancer. Molecules that inhibit the EIF4E–EIF4G interaction, such as 4EGI‐1, have showed promising effects in preclinical trials.^39^ The EIF4E‐EIF4G complex can be regulated by 4EBP1. 4EGI‐1, as a pharmacologically mimic 4EBP1, is developed to disrupt EIF4E‐EIF4G association. In addition, EIF4A‐EIF4G complex has been shown to involve in regulation of translational initiation. Tumour suppressor PDCD4 competes with EIF4A for binding to EIF4G1, as such inhibiting the initialization of translation.^40^ Another small molecule SBI‐0640756 (SBI‐756), as a first‐in‐class inhibitor targeting EIF4G1 and disrupting the EIF4F complex, showed inhibitory effects on tumour growth.^41^ We believe these kinds of molecules will be pharmacologically developed in the near future for clinical application to treat NSCLC patients.

In conclusion, the current study indicated that upregulation of EIF4G1 could activate the mTOR signalling pathway, thereby promote the tumour progression in NSCLC. These findings provide a new potential molecular target for the diagnosis and/or treatment of NSCLC patients.

## CONFLICT OF INTEREST

The authors declare that they have no conflicts of interest with the contents of this article.

## AUTHOR CONTRIBUTIONS

**Ying Lu:** Conceptualization (equal); Data curation (equal); Formal analysis (equal); Funding acquisition (equal); Supervision (equal); Writing‐original draft (equal). **Shanshan Yu:** Investigation (equal). **Guangxue Wang:** Methodology (equal). **Zuan Ma:** Methodology (equal). **Xuelian Fu:** Formal analysis (equal). **Yueyu Cao:** Data curation (equal). **Qinchuan Li:** Project administration (equal). **Zengguang Xu:** Conceptualization (equal); Funding acquisition (equal).

## Supporting information

Figure S1

## Data Availability

The data sets used during the present study are available from the corresponding author upon reasonable request.
