# Supplementary figures and images for: Elevation of EIF4G1 promotes non‐small cell lung cancer progression by activating mTOR signalling
Source: J Cell Mol Med. 2021 Feb 1;25(6):2994–3005. doi: 10.1111/jcmm.16340 (PMC7957198; doi:10.1111/jcmm.16340)

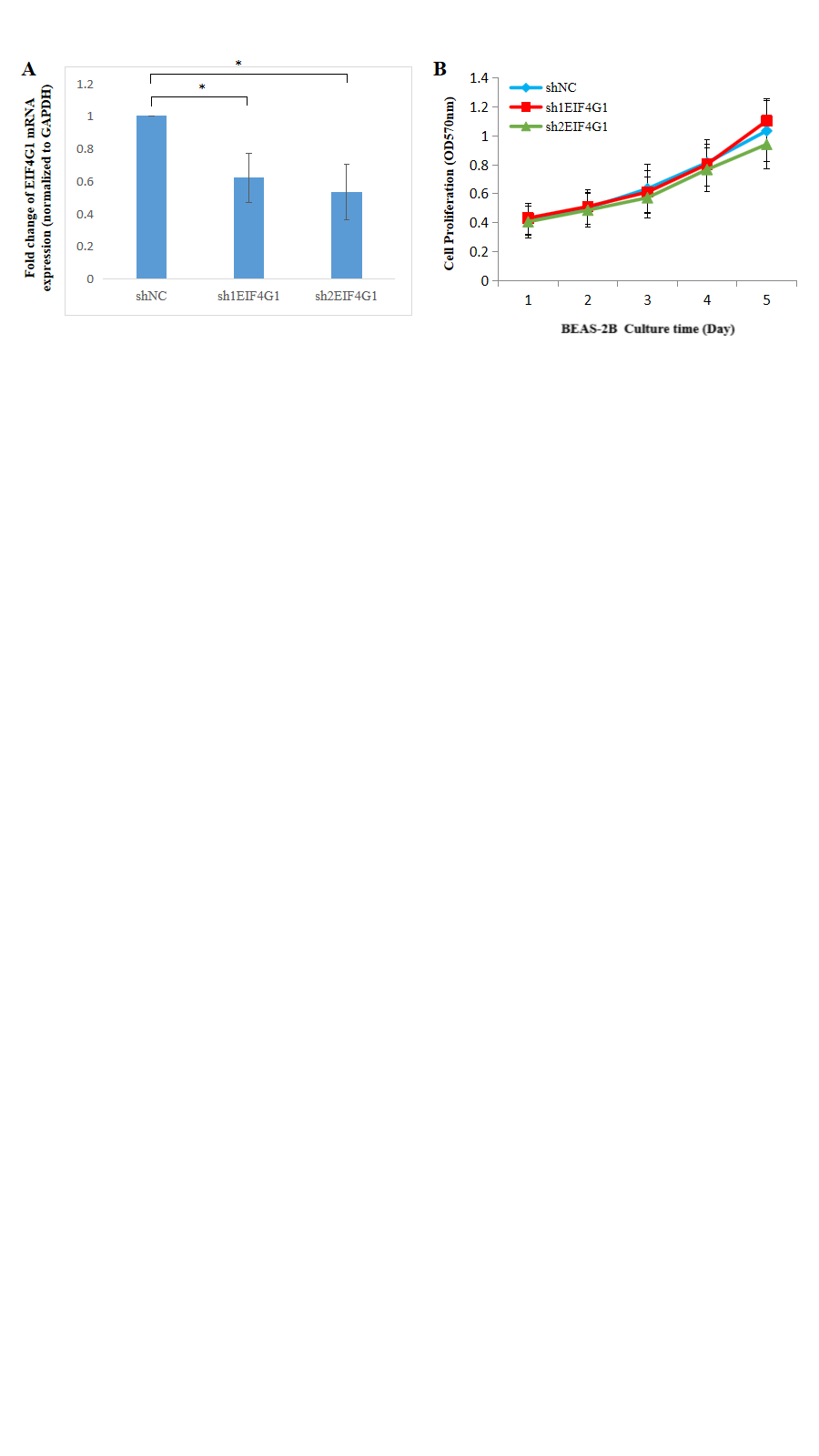

Supplement: Supplementary file 1 — Figure S1 [file JCMM-25-2994-s001.tif]
